# Dually Diagnosed Patients with Arrests for Violent and Nonviolent Offenses: Two-Year Treatment Outcomes

**DOI:** 10.1155/2016/6793907

**Published:** 2016-03-28

**Authors:** Christine Timko, Andrea Finlay, Nicole R. Schultz, Daniel M. Blonigen

**Affiliations:** ^1^Center for Innovation to Implementation (Ci2i), VA Palo Alto Health Care System, Palo Alto, CA 94304, USA; ^2^Stanford University School of Medicine, Stanford, CA 94305, USA; ^3^Department of Psychology, Auburn University, Auburn, AL 36849, USA; ^4^Palo Alto University, Palo Alto, CA 94304, USA

## Abstract

The purpose of this study was to examine the history of arrests among dually diagnosed patients entering treatment, compare groups with different histories on use of treatment and mutual-help groups and functioning, at intake to treatment and six-month, one-year, and two-year follow-ups, and examine correlates and predictors of legal functioning at the study endpoint. At treatment intake, 9.2% of patients had no arrest history, 56.3% had been arrested for nonviolent offenses only, and 34.5% had been arrested for violent offenses. At baseline, the violent group had used the most outpatient psychiatric treatment and reported poorer functioning (psychiatric, alcohol, drug, employment, and family/social). Both arrest groups had used more inpatient/residential treatment and had more mutual-help group participation than the no-arrest group. The arrest groups had higher likelihood of substance use disorder treatment or mutual-help group participation at follow-ups. Generally, all groups were comparable on functioning at follow-ups (with baseline functioning controlled). With baseline arrest status controlled, earlier predictors of more severe legal problems at the two-year follow-up were more severe psychological, family/social, and drug problems. Findings suggest that dually diagnosed patients with a history of arrests for violent offenses may achieve comparable treatment outcomes to those of patients with milder criminal histories.

## 1. Introduction

An increased risk for violent crime has been identified among individuals with dual substance use and mental health disorders, compared with general populations or persons with only mental health diagnoses [1–3]. However, little is known about how dually diagnosed individuals with a history of violent crime fare when they enter treatment. To fill this gap, this study compared three groups of dually diagnosed patients: those arrested for violent offenses, those with a history of arrest for nonviolent offenses only, and those with no history of arrest. The groups were compared on treatment and mutual-help group utilization and functioning. Comparisons were made at intake to treatment and over a two-year follow-up period.

*Arrest History and Use of Treatment and Mutual-Help Groups.* Research suggests that significant proportions of treated mental health patients report a history of having been arrested for violent and especially nonviolent offenses. This history is associated with more risk of hospitalization, but also more participation in mutual-help groups. In a large random sample of people drawn from the public mental health system in Los Angeles County, patients' claims were matched to criminal justice records over a 10-year period. Over the study period, 24% had at least one arrest; of arrested individuals, 38% had committed violent offenses (9% of the overall sample), and 62% had only nonviolent offenses. Individuals who had never been arrested did not differ from individuals with an arrest history on outpatient mental health treatment services received. Arrested persons had longer hospitalizations (4.1 versus 1.8 days) than persons who were not arrested [4]. Although this study did not describe participants' substance use diagnoses, drug-related crimes were the second most common type of offense after violent offenses.

In a large sample of offenders released from prison and referred to substance use disorder (SUD) treatment programs, 61% were dually diagnosed, and 27% had at least one violent offense during the six-month period preceding the arrest that led to their incarceration. Comparisons of participants with and without violent offenses found that the violent group had more psychiatric hospitalizations (lifetime), but there was no difference on previous SUD treatments [5].

A study of SUD patients identified predictors of greater participation in continuing care and mutual-help groups during the three months following completion of an intensive outpatient SUD program. More severe legal problems at the time of completion of the intensive program predicted more mutual-help group participation (but not more treatment utilization) at the level of a trend [6]. Similarly, SUD patients referred to outpatient treatment by the criminal justice system who had more arrests (mean number of arrests, lifetime = 14.0) were more likely to be frequent attenders of mutual-help groups (at least 3 meetings per week) than patients with fewer arrests (M = 7.3 arrests [7]). In contrast, among patients with both serious mental illness and SUD, those who had been arrested in the past 90 days were less likely to engage with SUD treatment (i.e., attend at least three outpatient sessions [8]).

*Treated Patients' Arrest History and Functioning.* The few studies available indicate that dually diagnosed patients with more serious arrest histories may also have poorer functioning. In the study of offenders released from prison and referred to SUD treatment programs, the group with a violent offense history had higher rates of mental health problems (lifetime depression; anxiety; difficulty in understanding, remembering, and concentrating) and more severe alcohol and drug problems at treatment intake [5]. Among homeless persons with severe mental illness entering a community treatment (case management) program, those with a history of longer incarceration had more severe substance use and psychiatric problems than those reporting no or shorter incarcerations. At one year after entry, patients with short- and long-term incarceration histories had more severe psychiatric problems than those never incarcerated [9].

*Correlates of Improved Legal Functioning*. Treated patients with improved functioning in terms of substance use, mental health, and other life problems may also have decreases in criminal behavior and legal problems. Among patients in a Department of Veterans Affairs (VA) Opiate Substitution Therapy Program, 47% reported arrests for violent crimes, 20% for nonviolent crimes only, and 16% for unspecified crimes; 17% had no arrest history [10]. Most of these patients, but especially those arrested for violence, perceived their drug treatment as helpful in addressing their criminal behavior. These findings agree with those of dually diagnosed male offenders who had a greater reduction in risk of committing new crimes after receiving SUD treatment compared to a reference category of those not receiving any SUD treatment [11].

Dually diagnosed Veterans' rates of having been charged with a crime were 11% in the year prior to receiving VA behavioral health services and 7% in the year after receiving services (a statistically significant decrease [12]). An 18-month study of individuals entering sober living houses, designed to provide appropriate housing to support sustained recovery, found that 42% had been arrested in the six-month period preceding intake. Participants reduced their alcohol and drug problem severity over time and were less likely to be arrested over the next three six-month periods of follow-up (26%, 22%, and 28% arrest rates, resp.). Residents referred to the houses by the criminal justice system, suggesting a more severe arrest history, had higher rearrest rates [13]. Similarly, jailed, seriously mentally ill adults randomly assigned to case management rather than usual care improved more on substance abuse and criminal activity [14].

*Present Study*. The literature reviewed suggests that a history of violent arrests among dually diagnosed individuals may be associated with a higher likelihood of hospitalization and use of mutual-help groups and poorer functioning in the mental health and substance use domains. Improvement in these domains may be associated with reduced criminal behavior and less legal system involvement. However, there have not been previous longitudinal studies comparing treated dually diagnosed patients based on their history of arrests for violent offenses. The purpose of this study was to (1) identify the rates of no arrest history, history of arrests for nonviolent offenses only, and history of arrests for violent offenses, among dually diagnosed patients entering treatment, (2) compare the three groups on treatment and mutual-help group utilization and functioning at baseline (intake to treatment) and six-month, one-year, and two-year follow-ups, and (3) examine correlates and predictors of legal functioning at the endpoint (the two-year follow-up). The goal was to inform treatment and criminal justice system managers and frontline staff regarding factors associated with improved outcomes among dually diagnosed patients with varying histories of violent crime.

## 2. Method

### 2.1. Sample and Procedure

At baseline, we collected data from 304 dually diagnosed patients. The sample was enrolled using the following procedure: patients entering outpatient mental health treatment and identified by program staff as having dual substance use and psychiatric disorders (based on the program's standard assessment procedures and medical record review) and as cognitively competent to provide informed consent were invited to participate in the study. Data were collected as part of an ongoing evaluation of treatment for dually diagnosed patients. Outpatient treatment was oriented toward the delivery of evidence-based practices (e.g., cognitive-behavioral, mindfulness approaches) in multidisciplinary individual and group therapy sessions that emphasized teaching skills such as symptom or stress management and relapse prevention. Out of 343 consecutive patients approached about the study, 39 refused to participate. Stanford University's Institutional Review Board for Human Subjects in Medical Research approved all study procedures, and all participants signed an informed consent form. According to the medical record, psychiatric diagnoses were mainly a major depressive or another mood disorder (60.5%) and PTSD (34.9%), and most participants had both alcohol and drug use disorders (65.5%).

Follow-up rates were 81% (*n* = 238) at 6 months, 82% (*n* = 241) at 1 year, and 84% (*n* = 238) at 2 years, among participants not known to have died or be incapacitated. Participants followed up at 6 months were somewhat more educated at baseline than those who were not followed up (13.6 versus 12.9 years; *p* < .05). Participants followed up at 2 years were somewhat older at baseline than those who were not followed up at that time point (51.7 versus 49.0 years; *p* < .05). Otherwise, attrition at follow-ups was not associated with any demographic or clinical characteristic at baseline.

### 2.2. Measures

*Baseline.* Self-report data, including demographics, arrest history, previous treatment, and mutual-help group utilization, and functioning, were collected from patients at baseline by trained research assistants.

To assess* arrest history*, we used the Addiction Severity Index (ASI [15, 16]), which is a structured, 40-minute clinical research interview. Participants reported the number of times in their lifetime they had been arrested and charged for 18 types of offenses. Nonviolent felony arrests consisted of burglary, breaking and entering or larceny, drug offenses, parole or probation violations, arson, shoplifting or vandalism, forgery, weapons offenses, prostitution, disorderly conduct, vagrancy or public intoxication, driving while intoxicated, contempt of court, and major driving violations. Violent felony arrests consisted of those for rape, homicide, robbery, and assault [17].

We also used the ASI to assess* treatment utilization* (i.e., number of lifetime episodes of outpatient, and of inpatient or residential, treatment for alcohol problems, drug problems, and psychological or emotional problems). To assess* mutual-help group participation*, participants were asked if they had ever attended a 12-step group (no/yes) and were asked about the number of meetings they had ever attended, the number of steps they had worked, and their involvement in 12-step practices. Involvement was the sum of 14 items (0 = no, 1 = yes), for example, calling a 12-step group member for help, reading 12-step group literature, being of service (helping at meetings), and sharing honestly during meetings; Cronbach's alpha = .90 or higher at baseline and follow-ups.

To assess* functioning* at baseline, we used ASI composite scores in the legal, alcohol, drug, psychological, employment, family/social, and medical domains. In each area, questions focus on the number, extent, and duration of symptoms in the past 30 days [18]. ASI composites range from 0 to 1, with higher scores indicating poorer outcomes. To indicate functioning, we also used two ASI items asking if the participant currently lived with someone with an alcohol problem and with a drug problem (0 = no, 1 = yes). Finally, we used ASI items asking if the participant had undergone a significant period of time, not a direct result of alcohol or drug use, in which he or she had experienced trouble controlling violent behavior, serious depression, serious anxiety or tension, and trouble understanding, concentrating, or remembering (0 = no, 1 = yes).

*Follow-Ups.* Trained research assistants collected self-report data at 6-month, 1-year, and 2-year follow-ups by telephone. To assess outpatient and inpatient/residential treatment utilization, the participant's VA medical record was used. Specialized mental health care was distinguished from specialized SUD care, pertaining to utilization between intake and the 6-month follow-up, the 6-month and 1-year follow-ups, and the 1- and 2-year follow-ups. Specifically, numbers of outpatient sessions received, and numbers of days of inpatient or residential care received, were calculated.

Mutual-help group participation was assessed at follow-up as it was at baseline, that is, any attendance, number of meetings attended, number of steps worked, and extent of involvement. The 6-month and 1-year interviews asked about participation over the past 6 months; the 2-year interview asked about participation over the past year.

At follow-ups, the ASI was used to assess functioning (i.e., participants' legal, alcohol, drug, psychological, employment, family/social, and medical composite scores). Symptoms in the past 30 days were assessed with regard to trouble controlling violent behavior, serious depression, serious anxiety, and trouble understanding, concentrating, or remembering (0 = no, 1 = yes).

### 2.3. Analysis Plan

At baseline, we compared three groups: lifetime history of (1) no arrests, (2) one or more of nonviolent offense arrests only, and (3) one or more of violent offense arrests. We used analyses of variance (ANOVAs) for continuous outcome variables and chi-square tests for categorical outcome variables. At follow-ups, we compared the three groups' treatment and mutual-help group participation using ANOVAs and their functioning using analyses of covariance that controlled for the baseline value of the outcome under consideration. To examine correlates of legal functioning at the 2-year follow-up, we conducted multiple regressions in which groups' arrest status was controlled (0 = none, 1 = nonviolent, 2 = violent) in examining concurrent associations of ASI alcohol, drug, psychological, employment, family/social, and medical composite scores with the ASI legal composite score. We then conducted lagged regressions to examine associations of ASI composite scores at the 6-month and 1-year follow-ups with ASI legal functioning at 2 years, controlling for group status at baseline.

## 3. Results

At intake to treatment, only 9.2% (*n* = 28) of participants had no history of arrest; 56.3% (*n* = 171) had a history of arrest for only nonviolent offenses, and 34.5% (*n* = 105) had been arrested for violent offenses. Attrition at follow-ups was not associated with arrest history.

### 3.1. Baseline Comparisons

Comparisons at baseline of the three groups of patients—those with no arrest history or a history of nonviolent or violent arrests—are in Table 1.

*Demographics*. There were no significant differences among groups in demographic characteristics. Most patients were male, about one-half were white, less than one-half were employed, and relatively few were married. On average, patients were 51 years old and had completed about one year of college.

*Treatment and Mutual-Help Group Utilization.* As shown in Table 1, comparisons on numbers of outpatient episodes found that patients in the nonviolent arrest group had more drug treatment than patients in the no-arrest group. In addition, patients in the violent arrest group had more psychiatric treatment than patients in the no-arrest and nonviolent arrest groups. On numbers of inpatient/residential episodes, patients in both arrest groups had more drug and psychiatric treatment than patients in the no-arrest group.

In addition, patients in the nonviolent and violent arrest groups were more likely than patients in the no-arrest group to have ever attended mutual-help for their substance use disorder and were more involved in 12-step practices. Patients in the violent arrest group had worked more of the 12 steps than patients in the no-arrest group. We conducted additional analyses to determine whether amount of mutual-help group participation differed among the groups when only patients who had attended 12-step meetings were considered. When comparisons were conducted on number of meetings attended, number of steps worked, and involvement using only participants who had ever attended a meeting, patients in the nonviolent (M = 5.5, SD = 3.4) and violent (M = 5.3, SD = 3.6) groups were more involved in 12-step practices than patients in the no-arrest group (M = 2.5, SD = 3.0; *F* = 7.09, *p* < .001).

*Functioning.* As expected, patients in the two arrest groups had more severe legal problems (higher ASI composite scores) than patients in the no-arrest group. Patients in the violent arrest group had more severe problems (higher ASI composite scores) than patients in the no-arrest and nonviolent arrest groups in the alcohol, drugs, and psychological domains. They also had more severe problems than the no-arrest group in the family/social and employment domains and more severe problems than the nonviolent arrest group in the medical domain. In accordance with results for alcohol and drug problem severity, patients in the violent arrest group were more likely to live with someone having an alcohol and a drug problem than patients in the other two groups.

Also as expected, patients in the violent arrest group were most likely to report having had trouble controlling violent behavior in their lifetime. They were also more likely than the nonviolent group to report having had trouble concentrating and understanding. Patients in the two arrest groups were more likely than those in the no-arrest group to have had lifetime symptoms of serious depression.

### 3.2. Six-Month Comparisons

*Treatment and Mutual-Help Utilization.* At the six-month follow-up, patients in the three groups did not differ on number of mental health outpatient treatment sessions they had received (Ms, SDs = 21.9 (17.0) for no arrest, 21.0 (21.8) for nonviolent arrest, and 22.6 (25.5) for violent arrest groups, resp.; *F* = 0.13). Most of the patients in each group had received SUD treatment, but patients in the two arrest groups were more likely to have done so than patients in the no-arrest group (Table 2). However, the three groups did not differ on number of SUD outpatient sessions received (Table 2). The latter finding held when the comparison was conducted only for patients who received at least one SUD outpatient session (Ms, SDs = 13.1 (10.9), 14.3 (12.7), and 14.1 (11.9) for the no-arrest, nonviolent arrest, and violent arrest groups, resp.; *F* = 0.07).

The groups did not differ on whether they had obtained inpatient/residential mental health or SUD treatment, or on the number of days they had obtained such treatment (Table 2). In addition, the lack of differences held when only patients who had obtained inpatient/residential mental health, or SUD, treatment were considered in analyses; Ms and SDs for the no-arrest, nonviolent arrest, and violent arrest groups, respectively, were, for mental health days, 38.8 (31.6), 31.7 (39.9), and 38.0 (32.2), *F* = 0.22, and for SUD days, 24.0 (6.9), 53.6 (37.3), and 52.4 (37.7), *F* = 0.90.

Regarding mutual-help group utilization, patients in the two arrest groups were more likely to have attended a mutual-help group meeting and were more involved in 12-step practices than patients in the no-arrest group (Table 2). Patients in the nonviolent arrest group attended more meetings and worked more steps than patients in the no-arrest group. When analyses were conducted for number of meetings, number of steps worked, and involvement using only patients who had attended a meeting, there were no significant differences among groups (not tabled).

*Functioning*. At the 6-month follow-up, when baseline functioning was controlled, patients with violent arrests had more severe problems (higher ASI composite scores) than patients with no arrests in the psychological domain (Table 2). Otherwise, the three groups did not differ on functioning in the past 30 days (Table 2) at the six-month follow-up when baseline functioning was controlled.

### 3.3. One-Year Comparisons

*Treatment and Mutual-Help Utilization*. At the one-year follow-up, there were no differences among groups in outpatient, or inpatient, mental health or SUD treatment utilization (Table 3). The lack of differences held for numbers of outpatient sessions and inpatient days when only treatment utilizers were considered (not tabled).

Patients in the arrest groups were more likely to have attended a mutual-help group meeting and to be involved in 12-step practices. In addition, patients in the violent arrest group had worked more steps than patients in the no-arrest group. The greater involvement of the arrested groups held when the comparison was conducted only for patients who had attended a mutual-help group meeting during the follow-up period (Ms, SDs = 5.0 (3.2), 7.8 (3.3), and 8.1 (3.0) for the no arrest, nonviolent arrest, and violent arrest groups, resp.; *F* = 4.06, *p* < .05); however, the finding of working more steps did not hold.

*Functioning*. On functioning, there were no differences among groups in ASI composite scores or in past 30-day symptoms (Table 3).

### 3.4. Two-Year Comparisons

*Treatment and Mutual-Help Utilization*. At the two-year follow-up, there were no differences in rates or amounts of outpatient mental health treatment received (Table 4), even when only patients who received any such treatment were analyzed for comparison (not tabled). Patients in the arrest groups were more likely to receive outpatient SUD treatment, but they did not receive more of this treatment (Table 4), even when only those with any attendance were analyzed (not tabled). At the two-year follow-up, there were no differences among groups in receipt of inpatient/residential mental health or SUD treatment (Table 4), even when only utilizers were selected to analyze number of days of use (not tabled).

Patients in the arrest groups were more likely to attend a mutual-help group and be more involved in 12-step practices (Table 4). Patients with a history of nonviolent arrests attended more meetings than patients with no arrest history. However, differences among groups in number of meetings and involvement did not hold when only patients who had attended a meeting were used for comparison (not tabled).

*Functioning*. When baseline functioning was controlled, the only difference among groups was that the violent arrest group had more severe legal problems (higher ASI composite scores) than patients in the other two groups.

### 3.5. Predictors of Legal Functioning at Two-Year Follow-Up

At the two-year follow-up, when arrest status was controlled, concurrent analyses found more severe drug (*b* = .258, *p* < .001) and psychological (*b* = .138, *p* < .05) problems were associated with more severe legal problems. In addition, lagged analyses showed that when arrest status was controlled, more severe drug problems at the one-year follow-up were associated with more severe legal problems at two years (*b* = .274, *p* < .05). Additional lagged analyses showed that when arrest status was controlled, more severe psychological (*b* = .141, *p* < .05) and family/social (*b* = .334, *p* < .001) problems at six months were associated with more severe legal problems at two years.

## 4. Discussion

In this sample of dually diagnosed patients beginning an episode of VA outpatient mental health treatment, 91% had a history of at least one arrest. Of patients with an arrest history, 38% had been arrested for a violent offense. Compared to other samples of both VA and non-VA treated patients, the arrest rate in our sample is higher, but the rate of violent offenses is similar [4, 5, 10]. Possibly, interviews by research assistants not associated with the treatment program encouraged honest reporting of past arrests; patients may be inhibited from reporting arrest histories when seeking admission to a treatment program [10]. The similarity of rates of arrests for violent offenses between this VA sample and non-VA samples supports conclusions that Veterans' violent offender rates are not higher than those of non-Veterans [19].

### 4.1. Intake to Treatment

Similar to previous research comparing violent and nonviolent offenders and nonoffenders, this study of dual diagnosis patients found both differences and similarities between the violent and nonviolent groups at intake to treatment. At baseline, the violent arrest group was more severe than the nonviolent and no-arrest groups in that they had more outpatient treatment episodes for psychiatric problems and poorer functioning in the psychiatric, alcohol, and drugs domains; they were also more likely to live with someone with substance use problems. In addition, the violent arrest group was more severe than the no-arrest group by reporting poorer functioning in the employment and family/social domains and more lifetime serious depression. In contrast to these results showing the severity of the violent arrest group in particular, both of the arrest groups—violent and nonviolent—had more inpatient and residential treatment episodes for drug and psychiatric problems prior to the episode beginning at baseline. The two arrest groups were also more likely to have ever attended a mutual-help group and were more involved in 12-step practices than the no-arrest group.

Previous studies also reported violent and nonviolent offenders to be similar on some aspects of treatment and functioning but different on others. Offenders were more likely to have used mental health services prior to their first criminal sentence than a matched community group of nonoffenders, and violent offenders were more likely to have used mental health services than nonviolent offenders [20]. Among incarcerated individuals, violent and nonviolent offenders did not differ on psychological functioning but were more impaired than controls [21]. On the other hand, poorer psychological functioning was associated with having subsequently committed violent rather than nonviolent crimes among former psychiatric inpatients [22]. The use of mental health services for psychological problems prior to offending suggests that the period preceding crime perpetration may be characterized by deteriorating health and the perceived need for increased support [11, 23]. It also suggests that there are opportunities to prevent criminal activities and arrests with targeted therapeutic assessments and interventions [20, 24], perhaps by focusing on poor impulse control and other criminogenic risk factors such as antisocial traits [22, 25].

### 4.2. Treatment and Mutual-Help Group Utilization and Functioning at Follow-Ups

In keeping with their greater severity at baseline, at the 6-month follow-up, more patients in the violent and nonviolent arrest groups had obtained SUD outpatient treatment and attended a mutual-help group meeting than patients in the no-arrest group, and the arrested groups were more involved in 12-step practices. The higher likelihood of SUD treatment or mutual-help group attendance and involvement by the arrested groups continued at the one- and two-year follow-ups. Both treatment providers and patients themselves appear to have responded appropriately to the greater severity of the arrested groups [26]. A previous study of SUD rather than dually diagnosed patients found that patients with a history of difficulty controlling violent behavior received more treatment and participated more in 12-step groups compared to nonviolent patients [27]. In addition, violent patients benefited more from 12-step group participation than nonviolent patients did. Such findings suggest that treatment providers should consider referring patients with a history of violence to 12-step groups to enhance the likelihood of recovery.

### 4.3. Functioning at Follow-Ups

The violent arrest group had poorer psychological functioning than the no-arrest group did at six months after intake and poorer legal functioning than the no-arrest and nonviolent arrest groups at two years after intake. However, generally, the three groups were comparable on functioning at follow-ups when baseline functioning was controlled. Our findings that dually diagnosed patients with a history of violent arrest were generally similar to initially less severe groups on treatment outcomes are encouraging in that treatment seemed to be responsive to the needs of patients reporting violence perpetration [27].

### 4.4. Predictors of Legal Functioning at Two-Year Follow-Up

When arrest status was controlled, milder drug and psychological problems at the two-year follow-up were associated with milder legal problems at the same time point [13, 14]. In addition, more severe psychological and family/social problems at six months and more severe drug problems at one year were associated with more severe legal problems at the two-year follow-up. A meta-analysis of offenders with mental health disorders also found that psychological, family, and substance abuse problems increased the risk of continued criminal activities [28]. Although mental illness and past violence are potent predictors of violent criminal recidivism and consequent legal problems, premeditated aggression in particular may be strongly linked to recidivism [29, 30]. Thus, assessment of premeditated aggression among dually diagnosed patients with a history of arrests for violent offenses may be useful for treatment planning in mental health and substance use disorder programs. In addition to mental health and substance use disorder treatment, assignment to mental health courts rather than traditional courts has been found to predict lower rates of recidivism and longer time to rearrest for violent and nonviolent offenders, although rearrest was still more common among individuals with a history of more severe offenses [31, 32].

## 5. Limitations

Although this study had a number of strengths, such as a large, unselected dually diagnosed sample with high follow-up rates over two years and the use of medical records to obtain treatment utilization data, it also had some limitations. All patients were treated within the VA, which is federally funded and operates the largest mental health treatment system in the US. Generally, VA health services are of similar quality and effectiveness to those in the private sector [33, 34]. However, the VA patient population has poorer health status compared with the general patient population [35–37]. The extent to which our findings will be replicated in studies of patients in other health care systems remains to be determined. The study was also limited by lack of variation in participants' mental health diagnoses (mainly depression and PTSD).

The use of an observational design, rather than a randomized controlled trial design, also has strengths and weaknesses. We could not randomly assign participants to different arrest histories, and we chose to not randomly assign participants with similar or different arrest histories to different types or amounts of treatment (or recommended mutual-help group participation) because our focus was on examining outcomes obtained in routine practice. We also chose to report outcomes reflecting short- (6 months), medium- (one year), and long-term (two years) follow-ups, to optimize the practical utility of the findings for treatment providers facilitating pathways to recovery and desistance. Such reporting is also in keeping with requirements for potential systematic reviews and meta-analyses in which results from more than one timepoint cannot be combined without a unit-of-analysis error. In addition, our data were based on self-report, which may provide opportunity for less-than-accurate self-disclosure. However, comparisons of self-reports of arrests and official records have concluded that self-reports are valuable for research [36, 37] and that self-reported arrest history data given by persons with mental health and substance use disorders are as valid as those provided by general offender populations [38]. Among individuals with these disorders, the reliability and validity of self-reported service utilization, when tested against record abstraction, have also been found to be satisfactory [39, 40]. Finally, we followed recommendations that all comparisons be reported without correction when there is consistency across results [41, 42]; however, there was the risk of spurious differences among groups when conducting multiple tests.

## 6. Conclusions

Addiction and mental health treatment providers recognize that they are involved in the risk management of patients who might pose a threat to public health and safety through engagement in violence [11, 43]. Although the groups representing varying arrest histories—for violent or nonviolent offenses, or no arrests—were comparable at follow-ups, we cannot be sure that risk management or other processes during treatment explain this finding. Nevertheless, our results suggest that dually diagnosed patients with a history of arrests for violent offenses achieve nearly equivalent outcomes to those of patients with less severe criminal histories when treatment takes place in “real-world” settings. However, our results also suggest that these treatment settings should consider promising adjunct approaches, such as Moral Reconation Therapy or other cognitive-behavioral therapies aimed at changing antisocial thinking [44], with dually diagnosed patients who entered the criminal justice system due to violence. Such approaches may help to reduce patients' engagement in crime and involvement with the legal system over the long term.

## Figures and Tables

**Table 1:** Baseline characteristics of dually diagnosed patients with no arrests (*n* = 28), nonviolent arrests (*n* = 171), or violent arrests (*n* = 105).

	Nonviolent		Violent arrests	*χ* ^2^/*F*
	No arrests	Arrests		
Demographics				
Male (%, *n*)	92.9 (26)	91.8 (157)	90.5 (95)	0.23
White (%, *n*)	53.6 (15)	50.3 (86)	45.7 (48)	0.80
Age (M, SD)	51.0 (9.5)	50.9 (9.6)	51.4 (7.3)	0.12
Years of education (M, SD)	13.9 (2.7)	13.5 (1.8)	13.4 (2.0)	0.52
Employed (%, *n*)	35.7 (10)	46.7 (79)	45.2 (47)	1.20
Married (%, *n*)	21.4 (6)	12.4 (21)	6.7 (7)	5.02
Treatment history: number of episodes (M, SD)				
Outpatient				
Alcohol	1.1 (1.9)	2.2 (2.5)	2.0 (2.5)	2.32
Drugs	0.8 (1.7)^a^	2.1 (2.4)^a^	1.8 (2.3)	3.70^*∗*^
Psychiatric	2.4 (1.8)^a^	2.4 (2.0)^b^	3.0 (2.5)^a,b^	3.01^*∗*^
Inpatient/residential				
Alcohol	2.4 (2.6)	2.4 (2.6)	3.0 (2.9)	1.42
Drugs	0.5 (1.1)^a,b^	1.7 (2.3)^a^	2.1 (2.5)^b^	5.33^*∗∗*^
Psychiatric	0.5 (1.1)^a,b^	1.7 (2.5)^a^	2.0 (2.4)^b^	3.91^*∗*^
Mutual-help group history				
Ever attended	85.7 (24)^a,b^	94.7 (162)^a^	99.0 (104)^b^	8.85^*∗∗*^
Number of meetings (M, SD)	95.8 (160.7)	535.5 (987.2)	491.0 (1,069.7)	2.25
Number of steps worked (M, SD)	3.0 (4.4)^a^	4.8 (4.4)	5.5 (4.8)^a^	3.30^*∗*^
Involvement (M, SD)	2.2 (2.9)^a,b^	5.2 (3.6)^a^	5.3 (3.6)^b^	8.92^*∗∗∗*^
Functioning				
ASI composites (M, SD)				
Legal	.008 (.038)^a,b^	.108 (.174)^a^	.113 (.180)^b^	4.59^*∗∗*^
Alcohol	.099 (.164)^a^	.145 (.200)^a^	.225 (.248)^a,b^	6.06^*∗∗*^
Drugs	.037 (.067)^a^	.063 (.077)^b^	.092 (.091)^a,b^	6.71^*∗∗∗*^
Psychological	.340 (.250)^a^	.382 (.192)^a^	.442 (.188)^a,b^	4.37^*∗∗*^
Employment	.663 (.244)^a^	.757 (.248)	.788 (.201)^a^	3.13^*∗*^
Family/social	.153 (.118)^a^	.219 (.173)	.259 (.185)^a^	4.49^*∗∗*^
Medical	.507 (.383)	.429 (.351)^a^	.525 (.360)^a^	2.46^*∗*^
Living with someone with (%, *n*)				
Alcohol problem	46.4 (13)^a^	57.6 (98)^b^	68.9 (71)^a,b^	5.99^*∗*^
Drug problem	42.9 (12)^a^	58.2 (99)^b^	67.0 (69)^a,b^	5.70^*∗*^
Symptoms, lifetime (%, *n*)				
Trouble controlling violence	50.0 (14)^a^	50.3 (86)^b^	71.4 (75)^a,b^	12.94^*∗∗*^
Serious depression	85.7 (24)^a,b^	96.5 (165)^a^	97.1 (102)^b^	5.19^*∗*^
Serious anxiety	89.3 (25)	92.4 (148)	96.2 (101)	2.47
Trouble understanding	82.1 (23)	66.7 (114)^a^	83.8 (88)^a^	11.38^*∗∗*^

Note: means that share a superscript are significantly different at *p* < .05;^*∗*^ *p* < .05, ^*∗∗*^ *p* < .01, and  ^*∗∗∗*^ *p* < .001.

**Table 2:** Six-month characteristics of dually diagnosed patients with no arrests (*n* = 26), nonviolent arrests (*n* = 134), or violent arrests (*n* = 78).

	Nonviolent		Violent arrests	*χ* ^2^/*F*
	No arrests	Arrests		
Treatment				
Outpatient				
Any SUD (%, *n*)	57.7 (15)^a,b^	79.1 (106)^a^	79.5 (62)^b^	5.37^*∗*^
Number of SUD sessions (M, SD)	7.0 (10.2)	10.4 (12.1)	9.9 (11.2)	1.05
Inpatient/residential				
Any MH (%, *n*)	14.3 (4)	23.4 (40)	18.1 (19)	1.95
Number of MH days (M, SD)	5.5 (17.4)	7.4 (23.4)	6.9 (19.9)	0.10
Any SUD (%, *n*)	11.5 (3)	23.1 (31)	20.5 (16)	1.98
Number of SUD days (M, SD)	2.6 (7.8)	11.6 (28.0)	10.0 (26.4)	1.42
Mutual-help groups				
Any attendance	46.2 (12)^a,b^	79.7 (106)^a^	80.5 (62)^b^	12.59^*∗∗*^
Number of meetings (M, SD)	17.5 (33.0)^a^	56.6 (86.4)^a^	47.2 (50.3)	3.26^*∗*^
Number of steps worked (M, SD)	1.2 (2.4)^a^	3.1 (3.7)^a^	2.9 (3.6)	3.04^*∗*^
Involvement (M, SD)	2.7 (3.6)^a,b^	6.6 (4.5)^a^	6.5 (4.4)^b^	8.95^*∗∗∗*^
Functioning				
ASI composites (M, SD)				
Legal	.012 (.029)	.054 (.122)	.036 (.110)	1.81
Alcohol	.103 (.133)	.101 (.168)	.109 (.194)	0.06
Drugs	.030 (.033)	.035 (.055)	.034 (.072)	0.09
Psychological	.121 (.049)^a^	.184 (.135)	.205 (.192)^a^	3.10^*∗*^
Employment	.666 (.254)	.644 (.294)	.656 (.305)	0.12
Family/social	.352 (.211)	.368 (.358)	.358 (.387)	0.11
Medical	.419 (.354)	.351 (.332)	.349 (.366)	0.58
Symptoms, past 30 days (%, *n*)				
Trouble controlling violence	7.7 (2)	9.7 (13)	15.6 (12)	2.01
Serious depression	46.2 (12)	59.7 (80)	56.4 (44)	1.64
Serious anxiety	50.0 (13)	64.2 (86)	67.9 (78)	2.66
Trouble understanding	65.3 (17)	53.2 (41)	50.7 (68)	1.91

Note: means that share a superscript are significantly different at *p* < .05. SUD: substance use disorder; MH: mental health; ^*∗*^ *p* < .05, ^*∗∗*^ *p* < .01, and ^*∗∗∗*^ *p* < .001.

**Table 3:** One-year characteristics of dually diagnosed patients with no arrests (*n* = 22), nonviolent arrests (*n* = 131), or violent arrests (*n* = 78).

	Nonviolent		Violent arrests	*χ* ^2^/*F*
	No arrests	Arrests		
Treatment				
Outpatient				
Any mental health (%, *n*)	86.4 (19)	84.0 (110)	89.7 (70)	1.42
Number of MH sessions (M, SD)	13.5 (15.0)	14.5 (20.2)	13.1 (17.9)	0.19
Any SUD (%, *n*)	36.4 (8)	51.9 (68)	50.0 (39)	1.84
Number of SUD sessions (M, SD)	4.6 (11.1)	7.5 (13.7)	7.1 (23.4)	0.33
Inpatient/residential				
Any mental health (%, *n*)	4.5 (1)	15.3 (20)	20.5 (16)	4.01
Number of MH days (M, SD)	1.0 (3.9)	5.1 (19.5)	6.1 (21.1)	0.76
Any SUD (%, *n*)	0.0 (0.0)	4.6 (6)	7.7 (6)	3.32
Number of SUD days (M, SD)	0.0 (0.0)	2.3 (13.9)	2.3 (13.3)	0.40
Mutual-help groups				
Any attendance	47.6 (10)^a,b^	71.8 (94)^a^	67.9 (53)^b^	4.58^*∗*^
Number of meetings (M, SD)	19.5 (33.7)	44.9 (68.5)	41.8 (72.4)	1.28
Number of steps worked (M, SD)	0.7 (1.6)^a^	2.6 (3.6)	2.8 (3.7)^a^	3.13^*∗*^
Involvement (M, SD)	2.1 (3.2)^a,b^	4.9 (4.5)^a^	4.8 (4.5)^b^	4.20^*∗*^
Functioning				
ASI composites (M, SD)				
Legal	.011 (.001)	.030 (.108)	.029 (.104)	0.29
Alcohol	.052 (.100)	.107 (.170)	.114 (.187)	1.16
Drugs	.014 (.021)	.034 (.062)	.032 (.064)	1.06
Psychological	.329 (.202)	.345 (.210)	.363 (.351)	0.40
Employment	.685 (.237)	.630 (.297)	.672 (.263)	1.05
Family/social	.112 (.057)	.162 (.117)	.172 (.123)	2.17
Medical	.368 (.339)	.378 (.363)	.402 (.358)	0.15
Symptoms, past 30 days (%, *n*)				
Trouble controlling violence	4.7 (1)	8.7 (11)	6.0 (5)	0.39
Serious depression	55.3 (12)	54.0 (71)	51.6 (40)	0.76
Serious anxiety	51.3 (11)	57.8 (76)	64.7 (50)	0.87
Trouble understanding	53.3 (11)	52.6 (69)	52.4 (41)	0.00

Note: means that share a superscript are significantly different at *p* < .05. SUD: substance use disorder; MH: mental health; ^*∗*^ *p* < .05.

**Table 4:** Two-year characteristics of dually diagnosed patients with no arrests (*n* = 21), nonviolent arrests (*n* = 135), or violent arrests (*n* = 80).

	Nonviolent		Violent arrests	*χ* ^2^/*F*
	No arrests	Arrests		
Treatment				
Outpatient				
Any mental health (%, *n*)	100.0 (21)	89.6 (121)	90.0 (72)	4.32
Number of MH sessions (M, SD)	27.4 (27.8)	23.2 (32.7)	23.0 (37.2)	0.20
Any SUD (%, *n*)	33.3 (7)^a,b^	60.7 (82)^b^	61.3 (49)^a^	5.93^*∗*^
Number of SUD sessions (M, SD)	3.8 (11.6)	13.4 (28.3)	15.9 (50.2)	1.21
Inpatient/residential				
Any mental health (%, *n*)	19.0 (4)	22.2 (30)	15.0 (12)	1.72
Number of MH days (M, SD)	5.0 (11.3)	7.8 (20.9)	3.7 (12.9)	1.84
Any SUD	4.8 (1)	8.9 (12)	7.5 (6)	0.51
Number of SUD days (M, SD)	0.3 (1.5)	2.3 (9.7)	2.6 (9.9)	0.68
Mutual-help groups				
Any attendance	38.1 (8)^a,b^	70.9 (95)^a^	67.5 (43)^b^	8.27^*∗*^
Number of meetings (M, SD)	16.1 (42.3)^a^	80.1 (137.1)^a^	60.7 (91.9)	2.90^*∗*^
Number of steps worked (M, SD)	2.1 (4.3)	3.6 (4.5)	3.2 (4.3)	1.14
Involvement (M, SD)	2.5 (3.8)^a,b^	6.2 (4.8)^a^	5.7 (4.8)^b^	5.32^*∗∗*^
Functioning				
ASI composites (M, SD)				
Legal	.006 (.001)^a^	.014 (.069)^b^	.047 (.136)^a,b^	3.17^*∗*^
Alcohol	.087 (.132)	.102 (.183)	.114 (.193)	0.24
Drugs	.030 (.070)	.029 (.058)	.026 (.065)	0.09
Psychological	.350 (.219)	.341 (.216)	.310 (.229)	0.63
Employment	.712 (264)	.628 (.301)	.679 (.286)	1.49
Family/social	.144 (.071)	.160 (.133)	.175 (.152)	0.52
Medical	.442 (.379)	.347 (.332)	.437 (.345)	2.31
Symptoms, past 30 days (%, *n*)				
Trouble controlling violence	9.5 (2)	11.3 (15)	16.3 (13)	1.30
Serious depression	38.1 (8)	47.5 (38)	50.7 (68)	1.22
Serious anxiety	61.9 (13)	58.2 (78)	58.8 (47)	0.10
Trouble understanding	57.1 (12)	50.0 (66)	43.8 (35)	1.47

Note: means that share a superscript are significantly different at *p* < .05. SUD: substance use disorder; MH: mental health; ^*∗*^ *p* < .05, ^*∗∗*^ *p* < .01.
